# Supplementary material for: Cryo-EM structures of human STEAP4 reveal mechanism of iron(III) reduction
Source: Nat Commun. 2018 Oct 18;9:4337. doi: 10.1038/s41467-018-06817-7 (PMC6194020; doi:10.1038/s41467-018-06817-7)
Supplement: Supplementary file 1 — Supplementary Information [file 41467_2018_6817_MOESM1_ESM.pdf]

Cryo-EM structures of human STEAP4 reveal mechanism of iron(III) reduction

Oosterheert et al.

Supplementary Information

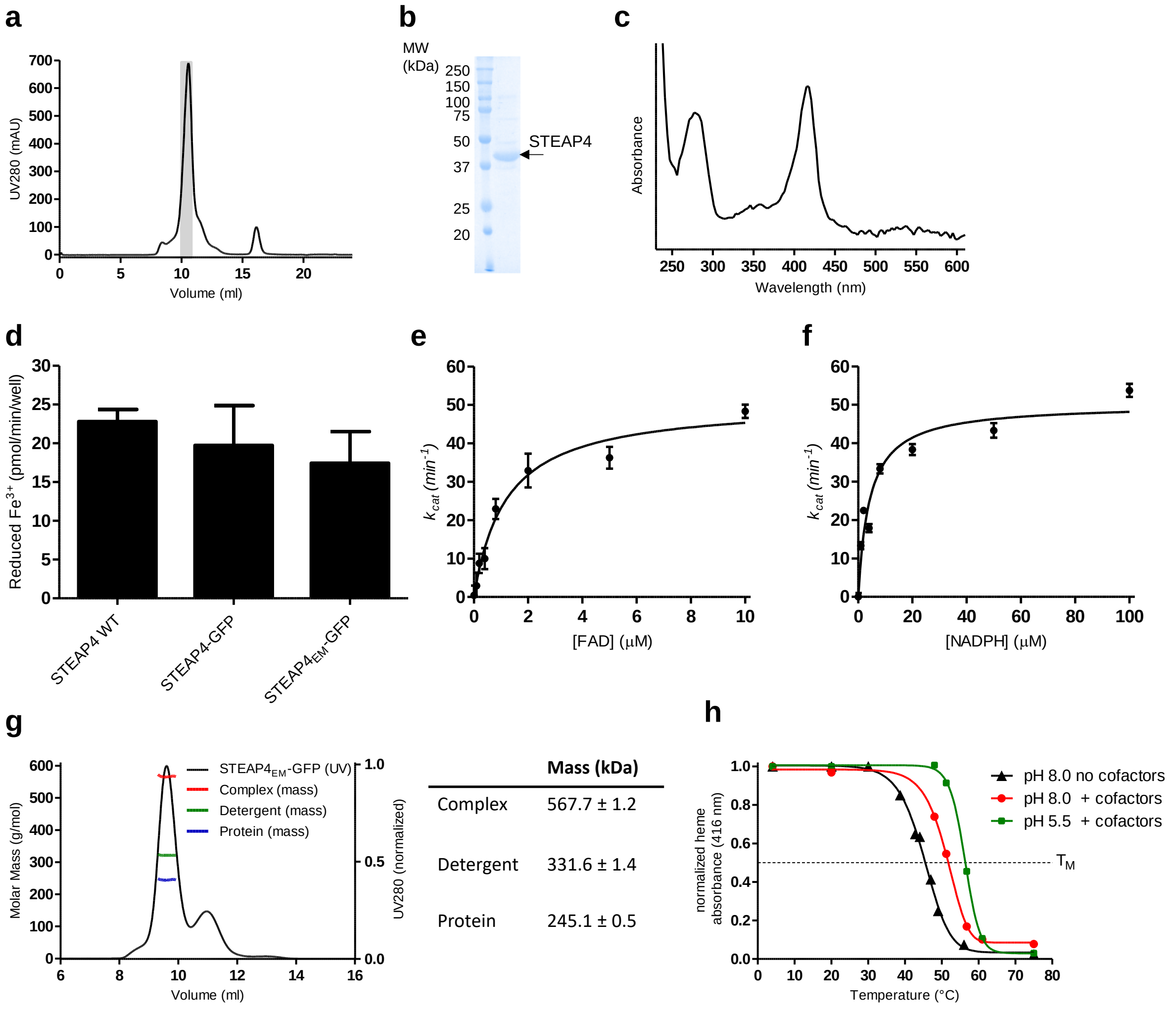

Supplementary Fig. 1. Biochemical characterization of human STEAP4.

(a) Size-exclusion chromatography elution profile of STEAP4$_{EM}$. The grey rectangle represents the collected fractions. **(b)** SDS-page gel of purified STEAP4$_{EM}$. **(c)** UV-Vis spectrum of purified STEAP4$_{EM}$. The heme-absorbance peak exhibits a maximum at 416 nm. **(d)** Cell-surface ferric-reductase activity of untagged STEAP4 (WT), GFP-Strep3-tagged STEAP4 and GFP-Strep3-tagged STEAP4$_{EM}$. The experiment was performed in triplicate. Error bars represent the standard error of the mean. **(e)** Ferric-reductase activity of purified STEAP4$_{EM}$ while varying the FAD concentration. STEAP4$_{EM}$ exhibits a K_d of 1.2 ± 0.2 μM for FAD. Data were fit to a single-site binding curve. Error bars represent the standard deviation between triplicate experiments. **(f)** Ferric-reductase activity of purified STEAP4$_{EM}$ while varying the NADPH concentration. STEAP4$_{EM}$ exhibits a K_M of 4.2 ± 0.7 μM for NADPH and a k_{cat} of 50.2 ± 2.1 min^{-1}. Error bars represent the standard deviation between triplicate experiments. **(g)** SEC-MALLS elution profile of GFP-tagged STEAP4$_{EM}$. The molar masses of complex (protein + detergent), detergent and protein are shown in red, green and blue respectively. The table reveals the calculated masses. The theoretical mass for a STEAP4$_{EM}$-GFP monomer is 82 kDa. **(h)** Melting curves for STEAP4$_{EM}$ at pH 8.0 or 5.5, in the absence of or presence of cofactors NADPH and FAD (both at 0.5-mM concentration).

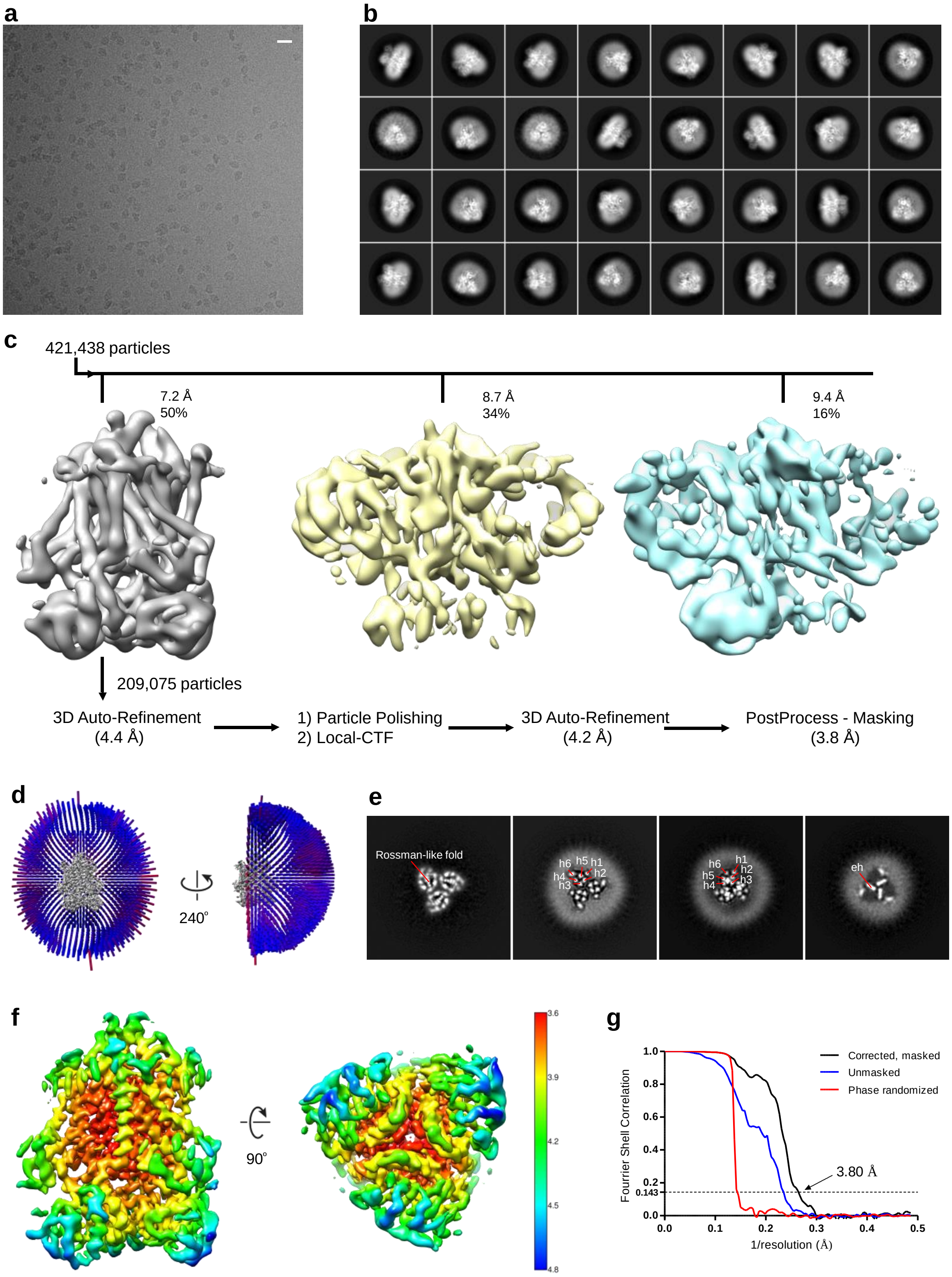

Supplementary Fig. 2. Cryo-EM data collection and processing for the cofactor-bound dataset.

(a) Micrograph of STEAP4$_{EM}$ particles in vitreous ice. The scale bar length is 200 Å. **(b)** Selected 2D classes of STEAP4$_{EM}$ particles generated by Relion[29]. **(c)** 3D classification of STEAP4 particles into 3 classes. Further classification of the unused classes did not yield maps

with protein features. **(d)** Angular distribution of particles included in the final reconstruction with C3 symmetry imposed. **(e)** 2D slices along the three-fold axis of the final unsharpened density map with annotated structural elements. **(f)** Local-resolution estimation of the final reconstructed map as determined by Relion. **(g)** Fourier-shell correlation (FSC) plot of high-resolution phase-randomized (red), unmasked (blue) and masked (black) half maps of $STEAP4_{EM}$. The FSC = 0.143 threshold is shown as a dashed line. The phase-randomized FSC curve was generated by Relion through a described procedure[1].

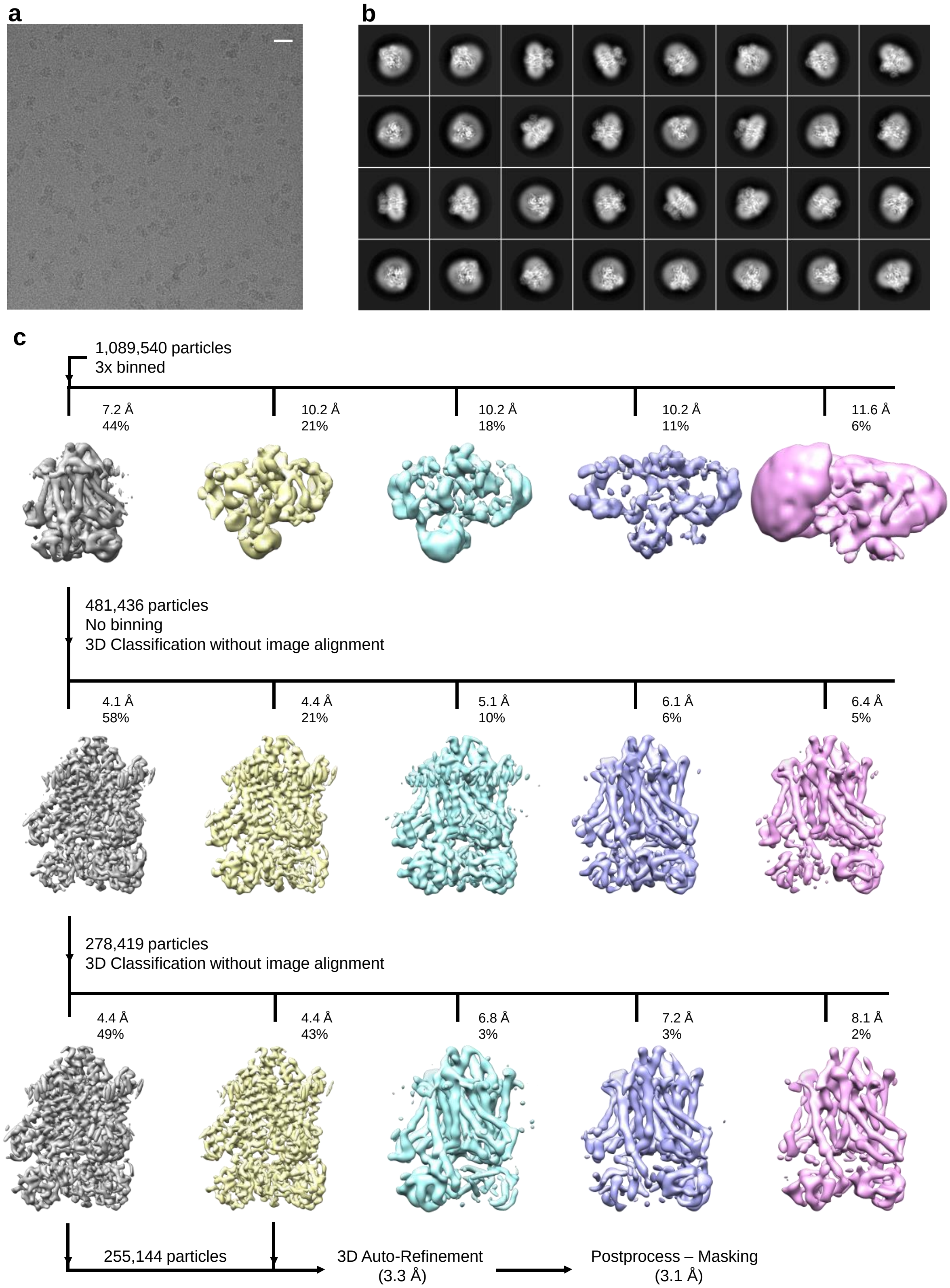

Supplementary Fig. 3. Cryo-EM collection and processing for the cofactor/substrate-bound dataset.

(a) Micrograph of $STEAP4_{EM}$ particles in vitreous ice. The scale bar length is 200 Å. **(b)** Selected 2D classes of $STEAP4_{EM}$ particles generated by Relion. **(c)** 3D classification (3 rounds) of STEAP4 particles into 5 classes. Further classification of the unused classes from the first round did not yield maps with protein features.

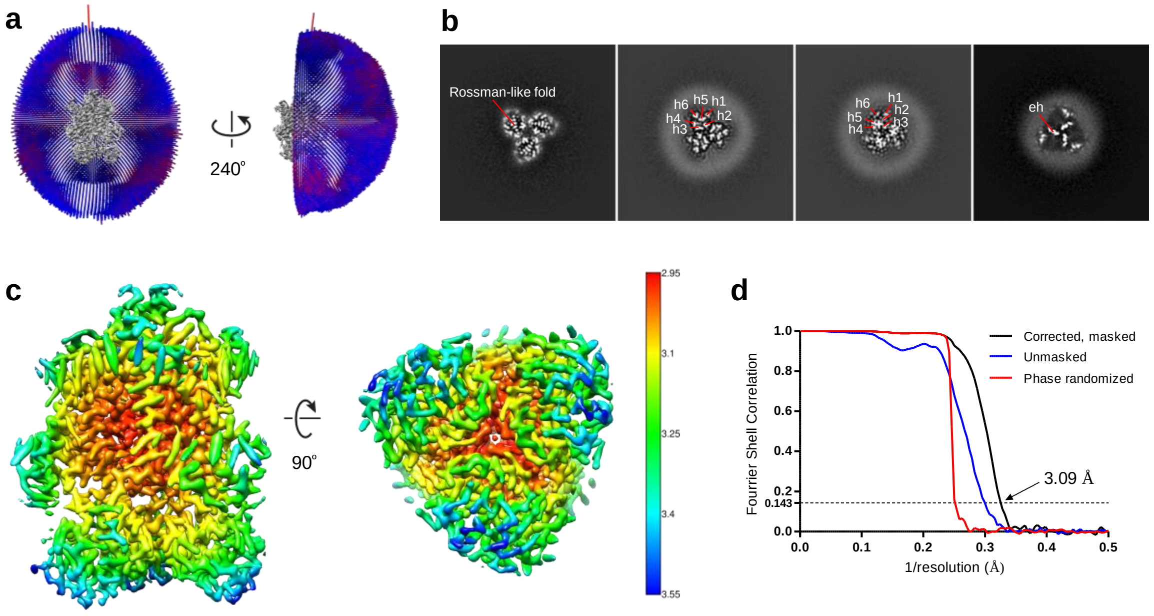

Supplementary Fig. 4. Cryo-EM collection and processing for the cofactor/substrate-bound dataset (continued).
(a) Angular distribution of particles included in the final reconstruction with C3 symmetry imposed. **(b)** 2D slices along the three-fold axis of the final unsharpened density map with annotated structural elements. **(c)** Local-resolution estimation of the final reconstructed map as determined by Relion. **(d)** FSC plot of high-resolution phase-randomized (red), unmasked (blue) and masked (black) half maps of $STEAP4_{EM}$. The FSC = 0.143 threshold is shown as a dashed line.

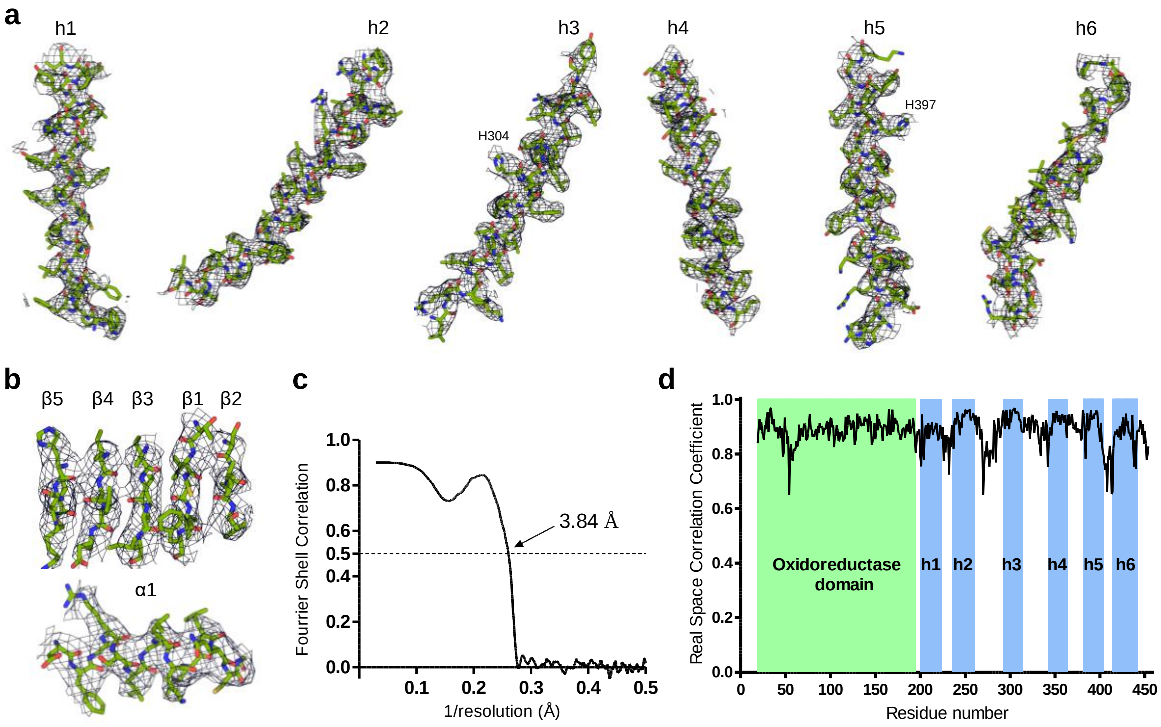

Supplementary Fig. 5. Modelling of cryo-EM map density in selected regions and model validation for the cofactor-bound structure.

(a) Density of all membrane helices with fitted amino acid residues. Density is contoured at 7.0σ. **(b)** Density and model for the first 5 parallel β-strands (contoured at 7.5σ) and the first α-helix (contoured at 6.5σ) of the intracellular OxRD. **(c)** FSC plot of the final reconstructed map versus the model as refined by Phenix[30]. The FSC = 0.5 threshold is shown as a dashed line. **(d)** Real-space correlation coefficient plotted for every amino-acid residue as calculated by Phenix. The intracellular OxRD and membrane helices are annotated.

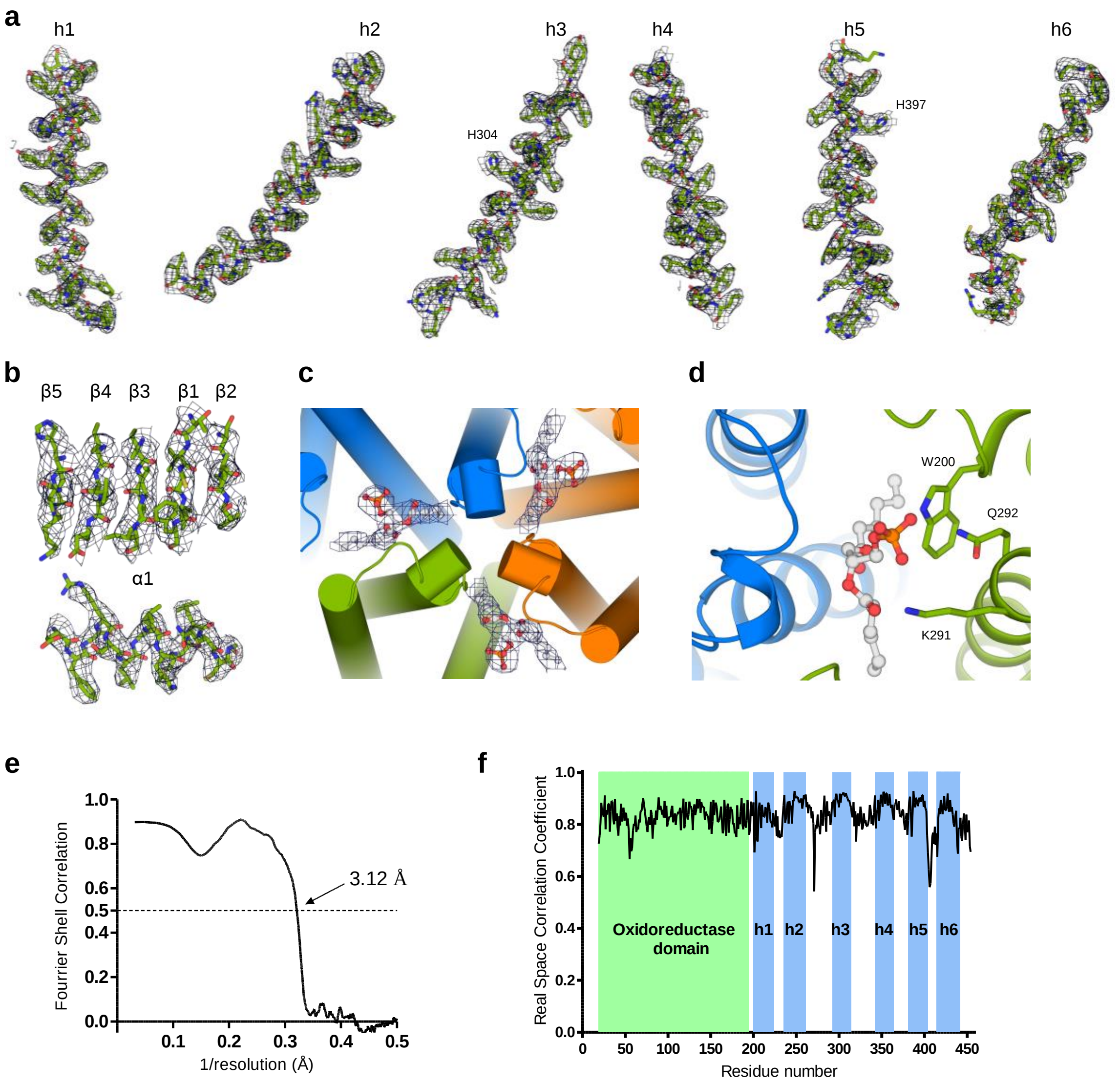

Supplementary Fig. 6. Modelling of cryo-EM map density in selected regions and model validation for the cofactor/substrate-bound structure.

(a) Density of all membrane helices with fitted amino acid residues. Density is contoured at 7.0σ. **(b)** Density and model for the first 5 parallel β-strands (contoured at 7.5σ) and the first α-helix (contoured at 6.5σ) of the intracellular OxRD. **(c)** Phospholipid packed between two STEAP4 subunits as viewed from the cytoplasmic site of the membrane. The intracellular OxRD and cofactor molecules are omitted for clarity. Due to the absence of clear headgroup density, the lipid was modeled as phosphatidic acid with fatty-acid chain lengths of 5 carbons. The density is contoured at 5.5σ. **(d)** Zoom of the lipid binding site. Residues W200, K291 and Q292 orient towards the lipid headgroup **(e)** FSC plot of the final reconstructed map versus the build model as determined by Phenix. The FSC = 0.5 threshold is shown as a dashed line. **(f)** Real-space correlation coefficient plotted for every amino-acid residue as calculated by Phenix. The intracellular OxRD and membrane helices are annotated.

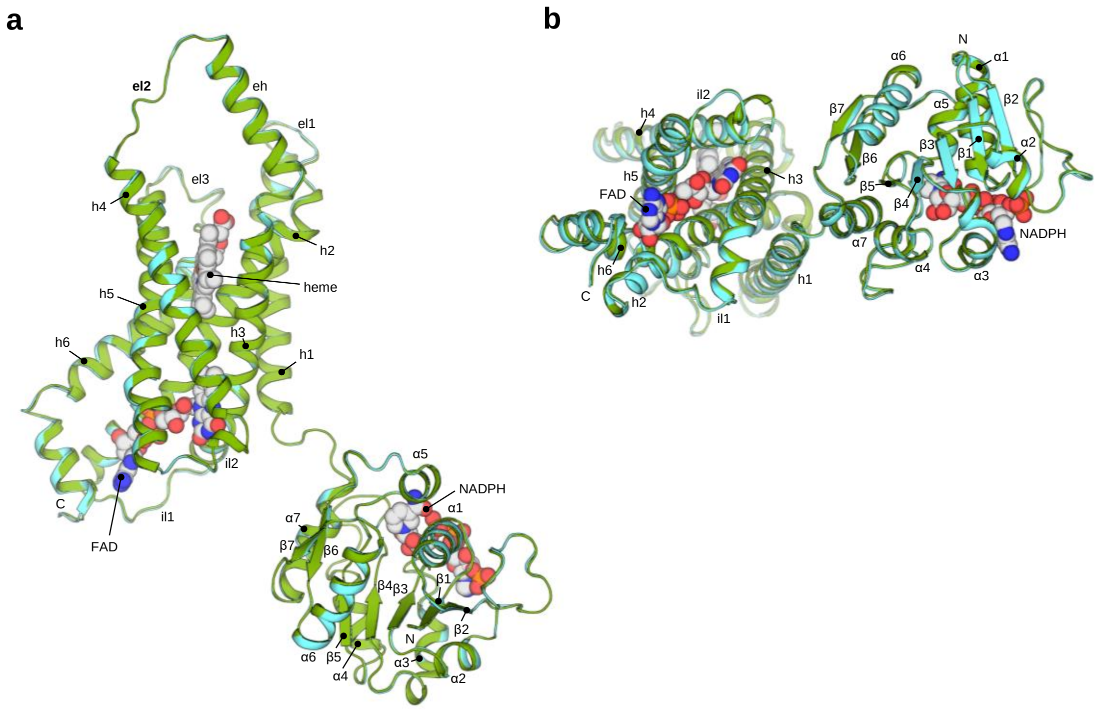

Supplementary Fig. 7. Superimposition and annotation of the cofactor-bound and cofactor/substrate-bound structures.

(a) ,**(b)** Superimposition of the monomeric cofactor-bound (cyan) and cofactor/substrate-bound (green) structures (rmsd = 0.3 Å for 436 Cα atoms) as viewed parallel to the membrane from the side **(a)** and orthogonal to the membrane from the cytoplasm **(b)**. Intracellular α-helices are annotated as α1-7, whereas membrane α-helices are defined as h1-6.

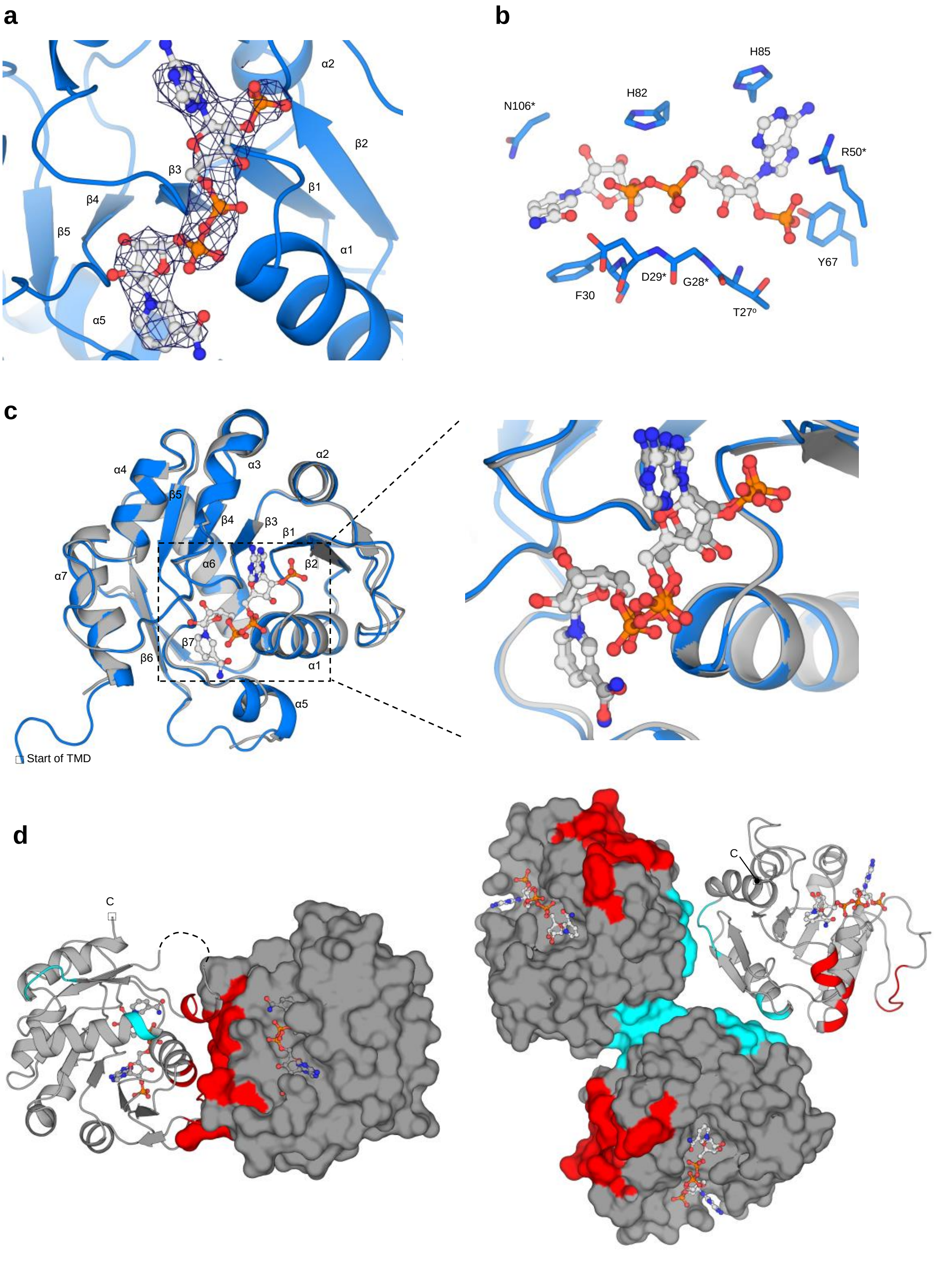

Supplementary Fig. 8. The intracellular oxidoreductase domain.
(a) Density for NADPH bound in the OxRD of each subunit. The density is contoured at 9σ. **(b)** Arrangement of NADPH-interacting amino acids. Residues annotated with (*) display >99% sequence conservation throughout all STEAP2-4 orthologs, whereas residues annotated with (°) exhibit conservative substitutions throughout all STEAP2-4 orthologs. **(c)** Superimposition of single-subunit OxRDs of human $STEAP4_{EM}$ (blue) and the isolated crystal structure of rat STEAP4 (grey), rmsd = 0.4 Å for 154 Cα atoms. **(d)** Comparison of the dimer as observed in the rat STEAP4 OxRD crystal structure[14] (left) and the trimeric OxRD

arrangement of $STEAP4_{EM}$ (right). The dimer interface is indicated in red in both structures; the trimer interface is colored cyan.

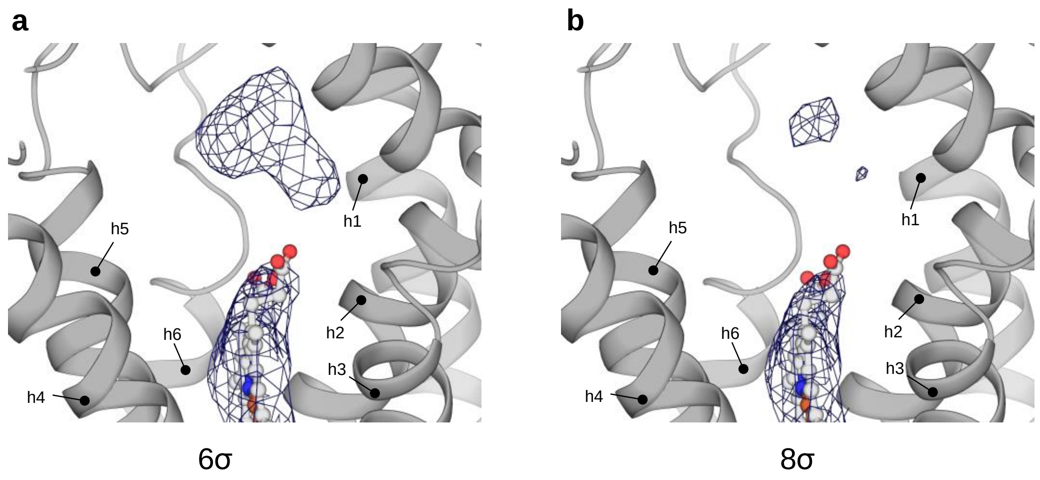

Supplementary Fig. 9. Substrate density in the unsharpened, non-difference map
(a) - (b) Densities of the heme cofactor and substrate contoured at 6σ and 8σ. The proposed substrate density is weaker than the density for heme.

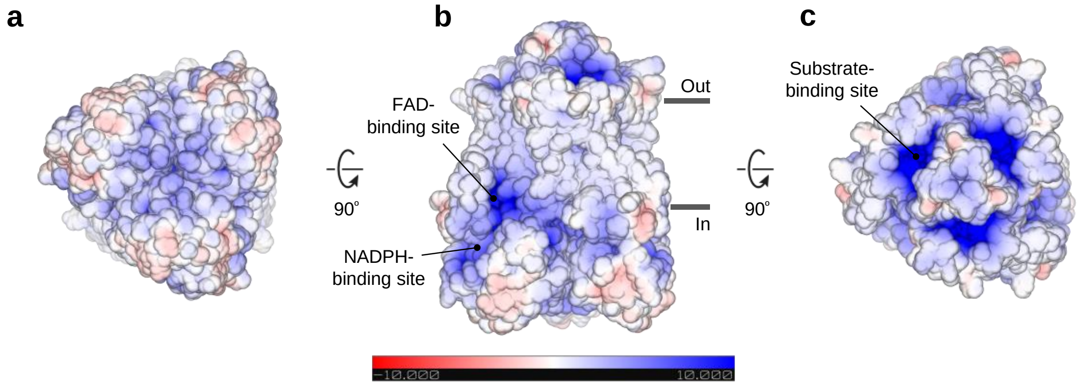

Supplementary Fig. 10. Electrostatic surface potential of STEAP4.
(a)-(c) Gradient visualization from red (-10 kT/e) to blue (10 kT/e) of the electrostatic surface potential of STEAP4 as viewed perpendicular to the membrane from the cytoplasm **(a)**, parallel to the membrane **(b)** and perpendicular to the membrane from the extracellular milieu **(c)**.

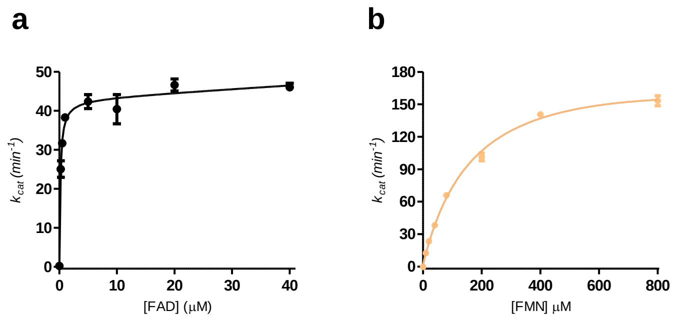

Supplementary Fig. 11. Catalytic activity of STEAP4 using different flavins as cofactor. **(a)** Ferric reductase activity of purified STEAP4 with varying FAD concentrations. A binding curve was fitted using non-linear regression in Graphpad Prism 5.0. GFP-tagged, full-length STEAP4 exhibits a K_d of 0.2 µM for FAD and a k_{cat} of 42.9 ± 0.8 min^{-1}. Error bars represent the standard deviation between triplicate measurements. The observed K_d values for FAD varied per STEAP4-batch but were always in the low micromolar range. **(b)** Ferric reductase activity of purified STEAP4 with varying FMN concentrations. A binding curve was fitted using non-linear regression in Graphpad Prism 5.0. GFP-tagged, full-length STEAP4 exhibits a K_d of 194 µM for FAD and a k_{cat} of 218 ± 20 min^{-1}. Error bars represent the standard deviation between triplicate measurements. We consider the high k_{cat} at extremely high FMN concentrations as an artefact.

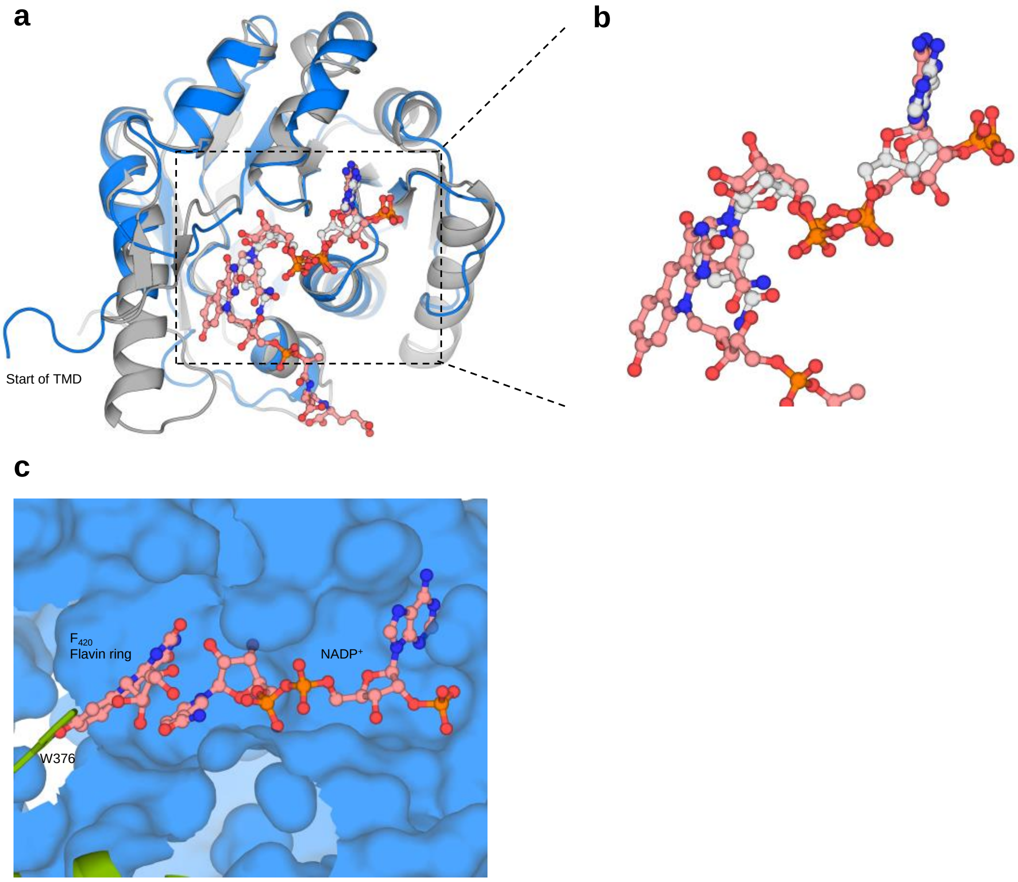

Supplementary Fig. 12. Structural comparison of the human STEAP4 OxRD to $F_{420}H_2$:NADP$^+$ oxidoreductase.

(a) Superimposition of a single-subunit OxRD of human STEAP4$_{EM}$ (blue) and the isolated crystal structure of FNO (grey, pdb = 1jay). **(b)** Zoom of the cofactor-binding site. NADP$^+$ bound to STEAP4 is coloured white, whereas NADP$^+$ and F_{420} of FNO are coloured pink. **(c)** Fit of the NADP$^+$-F_{420} arrangement of FNO in the structure of STEAP4. The F_{420} atoms that do not contribute to the interaction with NADP$^+$ are omitted from the figure. The flavin ring of F_{420} does not overlap with amino acids of the OxRD but clashes with conserved residue W376 from the loop between helices h4 and h5. We propose that a small reorientation of W376 would allow for efficient FAD-NADPH stacking.

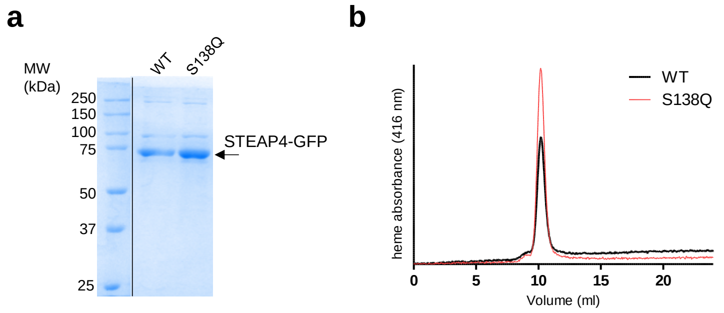

Supplementary Fig. 13. Purification of GFP-tagged full-length STEAP4 variants.
(a) SDS page gel of purified WT and S138Q variants of STEAP4. **(b)** Analytical size-exclusion chromatography elution profile of purified WT and S138Q variants of STEAP4.

Supplementary Table 1. Cloning and mutagenesis primers for STEAP4 constructs

**Cloning primers**			
Construct	Direction	Residue #	Sequence
$STEAP4_{EM}$	Forward	18	AATAATGGATCCAAGCAGGAAACCGTGTGC
$STEAP4_{EM}$	Reverse	456	AATAATGCGGCCGCGTTCCGCTCCCAGC
**Mutagenesis primers**			
Construct	Direction		Sequence
STEAP4 S138Q	Forward		TAACACCATCCAAGCCTGGGCCC
STEAP4 S138Q	Reverse		AAGGCCTTCACGACATGG

Supplementary References

1. Chen, S. *et al.* High-resolution noise substitution to measure overfitting and validate resolution in 3D structure determination by single particle electron cryomicroscopy. *Ultramicroscopy* **135,** 24–35 (2013).
